# Impact of Chronic Pain on Use-Dependent Plasticity: Corticomotor Excitability and Motor Representation in Musicians With and Without Pain

**DOI:** 10.1007/s10548-023-01031-1

**Published:** 2024-01-18

**Authors:** Anna M. Zamorano, Enrico De Martino, Ainhoa Insausti-Delgado, Peter Vuust, Herta Flor, Thomas Graven-Nielsen

**Affiliations:** 1 Center for Neuroplasticity and Pain (CNAP), Department of Health Science and Technology, Aalborg University, Selma Lagerløfs Vej 249 | Aalborg, 9260 Gistrup, Denmark; 2 TECNALIA, Basque Research and Technology Alliance (BRTA), Donostia-San Sebastián, Spain; 3 Center for Music in the Brain, Dept. of Clinical Medicine, Aarhus University & The Royal Academy of Music Aarhus/Aalborg, Aarhus, Denmark; 4 Institute of Cognitive and Clinical Neuroscience, Medical Faculty Mannheim, Central Institute of Mental Health, University of Heidelberg, Mannheim, Germany

**Keywords:** Musculoskeletal pain, Use-dependent plasticity, Repetitive movements, Sensorimotor training, Chronic pain

## Abstract

Long-term musical training induces adaptive changes in the functional representation of the motor cortex. It is unknown if the maladaptive plasticity associated with chronic pain, frequently affecting trained musicians, may alter the use-dependent plasticity in the motor cortex. This study investigated the interaction between adaptive and maladaptive plasticity in the motor pathways, in particular how chronic pain influences long-term use-dependent plasticity. Using transcranial magnetic stimulation (TMS), corticospinal excitability was assessed by measuring the amplitude of the motor-evoked potential (MEP), area of the motor map, volume, and center of gravity of the first dorsal interosseous muscle in 19 pain-free musicians, 17 upper limb/neck pain chronic pain musicians, and 19 pain-free non-musicians as controls. Motor map volume and MEP amplitude were smaller for both pain-free and chronic pain musicians compared to pain-free controls (P < 0.011). No significant differences were found between musicians with and without chronic pain. These findings confirm that long-term musical training can lead to focalized and specialized functional organization of the primary motor cortex. Moreover, the adaptive use-dependent plasticity acquired through fine-motor skill acquisition is not significantly compromised by the maladaptive plasticity typically associated with chronic pain, highlighting the potential of long-term sensorimotor training to counteract the effects of chronic pain in the motor system.

## Introduction

A growing body of research has highlighted that the repetitive sensorimotor training associated with musical training changes the corticomotor pathways, including a more refined distribution and reduced size of the representational cortical motor maps (Pascual-Leone 2001; Lotze et al. 2003). However, it remains unclear how maladaptive plasticity interacts with the established adaptive plasticity resulting from long-term musical training, particularly the plastic changes associated with chronic pain, a musculoskeletal disorder that affects up to 80% of trained musicians during their careers (Kok et al. 2016).

Maladaptive plasticity refers to disadvantageous changes in brain structure and function that can occur as a result of injury, disease, or abnormal sensory experiences. Patients with chronic pain show altered function and organization of the motor maps in the primary motor (M1) cortices compared to pain-free individuals (Schabrun et al. 2015). Even pain lasting for a few days alters corticomotor excitability and the corresponding motor maps (De Martino et al. 2018). Therefore, chronic pain could potentially affect the adaptive changes related to long-term musical training within the motor pathways.

The present study aimed to investigate the effects of chronic pain on corticomotor excitability and motor map representations in trained musicians, one of the best models for assessing long-term use-dependent plasticity. Following the studies of Pascual-Leone (2001) and Lotze et al. (2003), we hypothesized that pain-free musicians would exhibit use-dependent plasticity in the corticomotor excitability of the first dorsal interosseous (FDI) muscle, as indicated by a refined motor map volume and area compared to pain-free non-musicians. Moreover, it was hypothesized that musicians with chronic pain would exhibit preserved motor-evoked potentials (MEPs), attributed to ongoing training, alongside an enlarged motor area due to movement compensations caused by chronic pain.

## Results

### Corticomotor Excitability and Maps

**Fig. 1:**
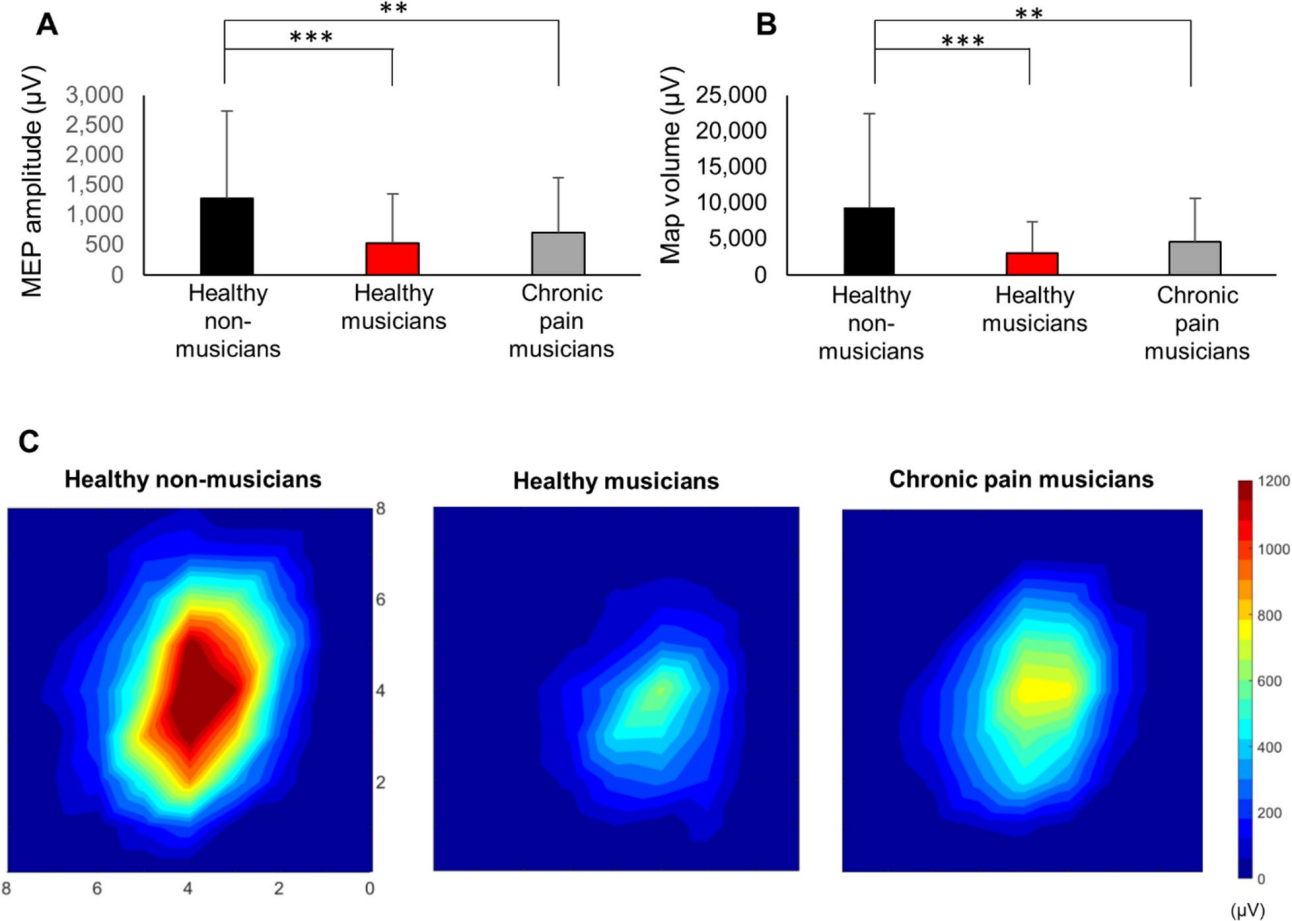
Corticomotor measures and maps in chronic pain musicians, pain-free musicians, and pain-free controls. **A** Reduced motor evoked potential (MEP) amplitude within the first dorsal interosseous (FDI) muscle map observed in both pain-free and chronic pain musicians. **B** Decreased map volume obtained for the right FDI muscle observed in both pain-free and chronic pain musicians. **C** Map representation obtained for the right FDI muscle. Coordinates are referenced to the stimulation site that evoked the greatest motor-evoked potential (center grid reference in the map) obtained for each individual. The X-axis represents the posterior side of the motor cortex, while the Y-axis represents the medial side of the motor cortex

The resting motor threshold (rMT), the mediolateral center-of-gravity (CoG) location of the motor map, and the number of discrete map peaks for the FDI muscle were not significantly different between groups (Table 1). However, map volume, map area, and MEP amplitude were significantly different between groups. Post-hoc tests indicated that the map volume (Fig. 1, Table 1) and maximum amplitude of an MEP within the map were reduced in pain-free musicians (*p* < 0.001; *BCa CI* 3824 to 9399 and *p* < 0.001; *BCa CI* 393 to 1103, respectively) and chronic pain musicians (*p* = 0.005; *BCa CI* 1839 to 7989 and *p* = 0.011; *BCa CI* 193 to 927, respectively) compared to pain-free controls. The map area was also reduced in pain-free musicians compared to pain-free controls (*p* = 0.004; *BCa CI* 3 to 12) but not significantly in chronic pain musicians compared to pain-free controls (*p* = 0.193; *BCa CI* −0.4 to 9). No significant differences were found between musicians with and without chronic pain in map volume and area, and MEP amplitude (all *p* > 0.517). No significant correlation was found between corticomotor excitability (RMT, MEPs, map area, and map volume) with the accumulated musical training and weekly training (all *p* > 0.05), possibly reflecting a potential ceiling effect. No significant correlation was found between corticomotor excitability (RMT, MEPs, map area, and map volume) with pain ratings or DASH questionnaire scores in chronic pain musicians (all p > 0.05).

**Table 1:** Corticomotor excitability measures and psychometric scores (mean ± SD) for chronic pain musicians, pain-free musicians, and pain-free controls

	Chronic pain musicians	Pain-free musicians	Pain-free controls	*F/t*	*p*	*Ƞ*^*2*^*/d*
*Corticomotor excitability*						
rMT (%)	40.7 ± 5.6	40.1 ± 6.4	40.3 ± 6.5	0.54	0.947	0.01
Map volume (mV)	4542 ± 3003*	2995 ± 2168*	9448 ± 6642	10.89	< 0.001	0.29
Map area (number of activated peaks)	15.2 ± 4.9	12.0 ± 5.8*	19.6 ± 9	5.71	0.006	0.18
Maximum MEP (µV)	705 ± 454*	525 ± 409*	1271 ± 732	9.31	< 0.001	0.26
CoG latitude (cm)	1.4 ± 2.1	1.0 ± 0.7	1.2 ± 0.6	0.39	0.674	0.01
CoG longitude (cm)	4.7 ± 0.6	4.6 ± 0.7	5.0 ± 0.6	2.79	0.070	0.10
Discrete peaks (n)	1.9 ± 0.8	1.5 ± 0.6	1.7 ± 0.6	1.31	0.279	0.05
*Psychometrics*						
DASH	13.3 ± 9.0	**2.4 ± 3.6#**	**3.3 ± 5.2#**	16.5	0.001	0.39
DASH music scale	19.1 ± 17.6	**2.3 ± 4.3#**	NA	4.04	0.001	1.37
s-STAI	10.8 ± 7.9	9.5 ± 8.3	12.4 ± 8.4	0.56	0.572	0.02
t-STAI	25.2 ± 8.8	24.0 ± 7.1	26.3 ± 8.2	0.36	0.699	0.01
PCS	14.6 ± 9.5	14.8 ± 8.3	15.4 ± 10.3	0.03	0.968	0.01
PVAQ	35.6 ± 11.1	26.4 ± 13.7	27.4 ± 11	3.15	0.051	0.11

*rMT* rest motor threshold; *MEP* motor evoked potential; *CoG* center of gravity; *DASH* Disabilities of the Arm, Shoulder and Hand; *PCS* pain catastrophizing scale; *PVAQ* pain vigilance and awareness questionnaire; *s-STAI and t-STAI* state-trait anxiety inventory; *NA* not applicable. *: Significant difference from pain-free controls (*,*p* < 0.05). **#**: Significant difference from chronic pain musicians (#, *p* < 0.001). Statistical parameters from the analysis of variance (*F*, *p*, effect size Ƞ^2^) or t-student (*t, d*)

### Psychometrics

Descriptive statistics were employed to provide insight into the pain experiences of the three groups. Notably, chronic pain musicians reported the highest levels of pain across all categories, including worst pain (5 ± 1.8), least pain (1.9 ± 1.7), average pain (3.8 ± 2.1), and current pain at the time of the experiment (2.7 ± 1.5). Pain-free musicians reported lower pain levels, with average ratings of 0.6 (± 1.2) for worst pain, 0 (± 0.0) for least pain, 0.1 (± 0.3) for average pain, and 0.0 (± 0.0) for current pain. Pain-free controls consistently reported the least pain, with all categories indicating minimal or no pain (worst pain: 0.0 ± 0.0, least pain: 0.0 ± 0.0, average pain: 0.4 ± 0.8, current pain: 0.0 ± 0.0).

One-way ANOVA revealed group differences on the Disabilities of the Arm, Shoulder and Hand DASH questionnaire (Table 1). Pain-free musicians and controls reported the lowest average DASH scores compared to chronic pain musicians (both *p* < 0.001), indicating upper extremity disability in this last group. The music scale yielded similar results, showing chronic pain musicians higher scores compared to pain-free musicians (*t* = 4.04; *p* < 0.001).

The scores of the State-Trait Anxiety Inventory and the Pain Catastrophizing Scale were not significantly different across groups (Table 1). However, there was a trend for the Pain Vigilance and Awareness Questionnaire scores to be higher in chronic pain musicians compared to pain-free musicians and pain-free controls (*p* = 0.074; Table 1).

## Discussion

This study examined the impact of chronic pain on the use-dependent plasticity of the motor cortex by exploring the corticomotor excitability of musicians with and without chronic pain. Pain-free musicians showed smaller corticomotor representation of the FDI muscle in the primary motor cortex compared to pain-free non-musicians. Previous research has shown that frequent practice of new motor skills leads to a rapid expansion in the volume and area of the motor representation and an increase in the amplitude of the corticomotor response of the muscles involved in that task (Pascual-Leone et al. 1995). However, with overlearning and automaticity of the task, as required for elite musical performance, this fine-motor skill acquisition leads to a reduction in the size of cortical maps due to the specialization and refinement of the structure and function of the motor cortex (Pascual-Leone 2001; Lotze et al. 2003). The current findings support these previous results and highlight that musical training can lead to the specialized functional organization of the primary motor cortex, as measured by corticomotor excitability.

Chronic pain did not significantly affect musicians' corticomotor excitability and functional motor representation of the FDI muscle, providing novel insights into how established use-dependent plasticity may act as a source of individual differences in pain processing. Neurobiological mechanisms such as metaplasticity, the plasticity of synaptic plasticity, may offer a framework for understanding the current results (Müller-Dahlhaus and Ziemann 2015; Altenmüller and Furuya 2016). Studies investigating the induction of metaplasticity have indicated that brief episodes of repetitive movement, preceding the application of non-invasive brain stimulation intended to induce LTP-like plasticity in the motor cortex, can alter the course of neurostimulation-like plasticity and lead to the emergence of LTD-like plasticity (Gentner et al. 2008; Iezzi et al. 2008). Chronic pain may disrupt neural mechanisms involved in metaplasticity such as homeostatic plasticity of the motor cortex (Thapa et al. 2018, 2021). Therefore, it is plausible that prior long-term musical training may have conferred neural stability to synaptic weights within motor pathways, enabling resistance and neuroprotective effects during chronic pain, which may have shaped the outcomes in chronic pain musicians. Following this hypothesis, previous studies have demonstrated that musicians with chronic pain exhibit lower interferences with daily activities and reduced insula-based connectivity compared to chronic pain non-musicians (Zamorano et al. 2019). Additionally, musicians report lower pain scores during the development of experimental muscle pain (Zamorano et al. 2023a). However, it is important to acknowledge the limitations of this study, such as the assessment of the cortical motor area (FDI) unrelated to the painful area (upper back) and the cross-sectional design, which may limit generalizability and causality establishment. Therefore, longitudinal studies employing more comprehensive methodologies for investigating the underlying mechanisms of corticomotor excitability in musicians coping with chronic pain should be carried out.

Althogether, the observed stability of corticomotor excitability in chronic pain musicians may reflect neuroprotective effects of extensive use-dependent plasticity associated to their ongoing musical training despite muscle pain. These findings underscore the potential impact of long-term sensorimotor training as a targeted strategy in chronic pain management programs.

## Materials and Methods

### Participants

A total of 55 participants were recruited through online advertising and flyers posted at Aalborg University, Aarhus University, and The Royal Academy of Music, Aarhus/Aalborg. Nineteen of these participants (eight female, 18 right-handed) were pain-free musicians trained in conservatories with a mean age of 26.6 ± 4 years and included 6 string players, 3 keyboardists, 7 woodwind players, and 3 brass players. They had a long history of professional experience with a total average of 17,600 (± 8,617) hours of musical practice and a current weekly practice average of 27.2 (± 14) hours. Seventeen participants were chronic pain musicians (thirteen females, 15 right-handed), with a mean age of 26.2 ± 4.3 years and an average pain history of 5 (± 4.7) years of chronic upper-limb (shoulder) or upper-back musculoskeletal pain syndromes. Chronic pain musicians were also conservatory-trained and had a long history of professional experience, with a total average of 17,020 (± 5,682) hours of musical practice and a current weekly average of 21.7 (± 6.8) hours. The group of chronic pain musicians was composed of three string players, three keyboardists, five woodwind players, and six brass players. All musicians were musically active at the time of the experiment. The accumulated training and weekly practice were obtained by interviewing the musicians and asking them to retrospectively recall the amount of practicing in different age periods (Zamorano et al. 2023b). The control group comprised 19 pain-free non-musicians (eight females, 18 right-handed) with a mean age of 26.9 ± 5.5 years. Exclusion criteria were neurological, cardiorespiratory, or mental disorders, pregnancy, as well as chronic pain in the case of pain-free musicians and controls. Participants completed a transcranial magnetic stimulation (TMS) safety screen (Keel et al. 2001) prior to commencement. The sample size was estimated based on previous publications using similar approaches (Schabrun et al. 2015) to ensure 80% power for detecting at least a medium effect size (Cohen’s d ≥ 0.6) with a one-way measures analysis of variance (ANOVA) at an alpha level of 0.05. All participants received written and verbal information about the study and provided written consent. The study was performed in accordance with the Declaration of Helsinki (General Assembly of the World Medical Association 2013) and approved by the local ethics committee (Den Videnskabsetiske Komité for Region Nordjylland, N-20170040).

### Experimental Procedure

The experiment consisted of a single session. Participants were seated comfortably in a chair and familiarized with the transcranial magnetic stimulation. Demographic information was collected, and participants completed psychometric measures assessing anxiety, catastrophizing, and vigilance to pain, and current pain ratings using a 0–10 numerical rating scale (NRS), with 0 defined as no pain and 10 defined as maximal pain. Neurophysiological testing, including motor cortical maps and corticomotor excitability, were performed.

### Transcranial Magnetic Stimulation

Single-pulse transcranial magnetic stimuli (TMS) were delivered using a figure-of-eight coil and a Magstim 200 stimulator (Magstim Co. Ltd.). The coil was placed over the left hemisphere at a 45-degree angle to the sagittal plane to preferentially induce current in a posterior-to-anterior direction. The optimal cortical site (the “hotspot”) to evoke responses in the right FDI muscle was determined as the coil position that elicited a maximal peak-to-peak motor evoked potential (MEP) for a given stimulation intensity. All TMS procedures adhered to the TMS checklist for methodological quality (Chipchase et al. 2012). Two measures of the cortical excitability were recorded during complete rest: (1) Resting motor threshold (rMT), defined as the minimum TMS intensity at which 5 out of 10 stimuli applied at the optimal scalp site evoked a response with a peak-to-peak amplitude of at least 50 μV. (2) Ten MEPs were recorded at 120% of rMT over the hotspot site to evaluate corticomotor excitability. MEP responses were measured as peak-to-peak amplitudes and averaged for analysis.

### Recording of Motor-Evoked Potentials

Electromyographic (EMG) activity was recorded from the muscle belly of the right First Dorsal Interosseus (FDI) using silver/silver chloride surface electrodes (Neuroline 720-01-K, Ambu® A/S). The EMG signals were sampled at 4 kHz, pre-amplified (1000 × gain), and bandpass filtered between 5 Hz and 2 kHz. The data were digitized by a 16-bit data acquisition card (National Instruments, NI6122) and saved using custom-made Labview software (Mr. Kick, Knud Larsen, SMI, Aalborg University).

EMG activity was preprocessed offline using Matlab (The Math-Works, Natick, USA). First, the TMS stimulation artefact was removed and the voltage difference at the extremities of the segments was corrected before being merged. The resulting signal was bandpass filtered between 5 and 1000 Hz and notch filtered using a 2nd-order Butterworth filter.

### Motor Cortical Maps

The cortical mapping procedure was done following a previous study (De Martino et al. 2018). A cap with a 1 × 1 cm grid was fitted in the head of participants and centered on the vertex (point 0, 0). TMS was applied every 6 s with 5 stimuli at each site, using a stimulus intensity of 120% rMT. The scalp sites were pseudo-randomly assessed until no MEP was recorded, defined as < 50 µV peak-to-peak amplitude in all 5 trials in all border sites. Trials with background EMG activity were excluded from the analysis.

The number of active map sites and map volume were calculated off-line, with a site considered “active” if the mean peak-to-peak amplitude of the 5 MEPs evoked at that site was greater than 50 µV. The map volume was calculated by summing the MEP amplitudes from the active sites. The map area was calculated by summing the number of active sites. The center of gravity (CoG) was defined as the amplitude-weighted center of the map and was calculated for each muscle using the formula:

$$CoG=\frac{\Sigma V* Xi}{\Sigma Vi},\frac{\Sigma Vi*Yi}{\Sigma Vi}$$

where Vi represents mean MEP amplitude at each site with the coordinates Xi, Yi.

### Psychometric Measures

All participants reported current pain ratings in resting conditions at the beginning of the lab session using a Numeric Rating Scale (NRS) with 0 defined as “no pain” to 10 corresponding to “worst pain imaginable”. In addition, NRS ratings for the worst, the least, and the average muscle pain during the previous week before the experiment were also reported.

Furthermore, to assess the disability and functional status of the upperlimb, all participants fulfilled the Disabilities of the Arm, Shoulder and Hand (DASH) questionnaire (Hudak et al. 1996). The DASH consists of 30 items which ask about the degree of difficulty in performing various physical activities because of an arm, shoulder, or hand problem (21 items), the severity of each of the symptoms of pain (5 items), and the effect of the problem on social activities, work, and sleep (4 items). The DASH also contains an optional 4-item scale concerning the ability to play a musical instrument (music scale), which was fulfilled by both groups of musicians. Each item is scored on a 5-point scale ranging from “no difficulty or no symptom” to “unable to perform activity or very severe symptom.” The scores for all items are used to calculate a final score ranging from 0 (no functional disability) to 100 (severe functional disability).

Anxiety was assessed using the State-Trait Anxiety Inventory (STAI) (Spielberger 2010), which contains two 20-item multiple-choice subscales measuring trait (personal quality) and state (situational) anxiety. Catastrophizing was assessed using the Pain Catastrophizing Scale (PCS) (Sullivan et al. 1995), which measures catastrophic thinking related to pain across three subscales: rumination, magnification, and helplessness. Vigilance to pain was assessed using the Pain Vigilance and Awareness Questionnaire (PVAQ) (Roelofs et al. 2003), a 16-item measure of attention to pain that assesses awareness, consciousness, and vigilance. The PVAQ consists of two subscales: “Attention to pain” (e.g., 'I pay close attention to pain') and “Attention to changes in pain” (e.g., 'I am quick to notice changes in pain intensity').

### Statistics

Data were presented as mean and standard deviation of the mean and were analyzed with IBM SPSS Statistics 27 for Windows. The normality and homogeneity assumptions were checked using Shapiro–Wilk’s, Mauchly’s, and Levene’s tests, as well as descriptive plots. Descriptive statistics were selected for analyzing NRS (Numeric Rating Scale) ratings due to the absence of variance in the pain-free and control groups. A one-way ANOVA was conducted to analyze all psychometric data (STAI, PCS, PVAQ, DASH), PPTs, and corticomotor excitability outcomes (rMT, maximum MEP within the map, map volume and area, number of active sites, latitude CoG and longitude CoG) across groups. Post-hoc comparisons were performed using Bonferroni. If the assumption of normality and homogeneity were violated, we used bootstrapping, a robust resampling technique that generates multiple datasets by sampling with replacement from the original data, and then analyzes these datasets to estimate the sampling distribution of a statistic. This approach provides more accurate p-values and confidence intervals, particularly when the data do not follow a normal distribution or when the sample size is small. The significance of the results was tested using bias-corrected and accelerated (BCa) 95% confidence intervals (CI), which adjust for possible bias and skewness in the bootstrap distribution. If zero was not within the 95% CI, we concluded that the indirect effect was significantly different from zero at *p* < 0.05. In both groups of musicians, correlations were used to investigate whether corticomotor excitability could be associated with the accumulated and weekly musical training. It was furthermore tested if the pain ratings and/or disability scores (DASH questionnaire) could have affected the corticomotor excitability in musicians with chronic pain. The level of significance for all tests was set at *p* < 0.05.

## Data Availability

The datasets generated and analysed during the current study are available from the corresponding author on reasonable request.
